# Generation of *mRx-Cre* Transgenic Mouse Line for Efficient Conditional Gene Deletion in Early Retinal Progenitors

**DOI:** 10.1371/journal.pone.0063029

**Published:** 2013-05-07

**Authors:** Lucie Klimova, Jitka Lachova, Ondrej Machon, Radislav Sedlacek, Zbynek Kozmik

**Affiliations:** 1 Institute of Molecular Genetics, Academy of Sciences of the Czech Republic, Prague, Czech Republic; 2 Department of Genetics and Microbiology, Faculty of Science, Charles University in Prague, Prague, Czech Republic; University of Regensburg, Germany

## Abstract

During mouse eye development, all retinal cell types are generated from the population of retina-committed progenitors originating from the neuroepithelium of the optic vesicle. Conditional gene inactivation provides an efficient tool for studying the genetic basis of the developing retina; however, the number of retina-specific Cre lines is limited. Here we report generation of the *mRx-Cre* BAC transgenic mouse line in which the expression of *Cre* recombinase is controlled by regulatory sequences of the mouse *Rx* gene, one of the earliest determinants of retinal development. When *mRx-Cre* transgenic mice were crossbred with the *ROSA26R* or *ROSA26R-EYFP* reporter lines, the Cre activity was observed in the optic sulcus from embryonic day 8.5 onwards and later in all progenitors residing in the neuroepithelium of the optic cup. Our results suggest that *mRx-Cre* provides a unique tool for functional genetic studies in very early stages of retinal development. Moreover, since eye organogenesis is dependent on the inductive signals between the optic vesicle and head surface ectoderm, the inductive ability of the optic vesicle can be analyzed using *mRx-Cre* transgenic mice.

## Introduction

Mammalian eye organogenesis is a multistep process of complex morphological events that involves interaction of the forebrain-derived optic vesicle (OV) with lens-competent head surface ectoderm (SE). If sufficient, this interaction leads to the coordinated invagination of both OV and SE resulting in formation of the optic cup (OC) [1]. The inner layer of OC is populated by retinal progenitor cells that further differentiate into seven retinal cell types: ganglion cells, amacrine cells, bipolar cells, horizontal cells, cone and rod photoreceptors and Muller glia cells. In previous three decades, several genes have been identified to play a crucial role in this process using the classical knockout strategies [2], [3], [4]. However, in some cases inactivation of such genes can lead to very early arrest of the eye development or even to the embryonic lethality, as can be demonstrated for the *Pax6* gene [5], [6]. This fact makes the dissection of cell autonomous function of a gene in early retinal progenitors of the OV and OC complicated. Nevertheless, the introduction of Cre-loxP-mediated tissue-specific gene inactivation can bypass embryonic lethality and allow functional study of the developmentally essential genes [7]. To perform conditional gene inactivation in retinal progenitors, utilization of a few *Cre* recombinase-expressing mouse lines has been reported. However, these lines display certain limitations and do not always offer sufficient strength at early stages [8], [9], [10], [11] or the required specificity [12], [13].

The homeodomain transcription factor *Rx* gene is one of the earliest genes expressed in the retinal lineage. It has been shown to be activated between embryonic day (E) 7.5 and E8.0 in the anterior neural plate and later strongly expressed in the optic vesicles and ventral forebrain [14], [15]. Rx function is essential for vertebrate eye development. Loss of the *Rx* function results in loss of eyes in various vertebrate species [14], [15], [16], [17], [18], [19], [20], suggesting its conserved role in the eye development. The early onset of *Rx* expression in the retinal primordium suggests that the *Rx* locus could be utilized for driving the *Cre* expression at very early stages of retinal development.

One such *Cre-*expressing line, taking advantage of *Rx* expression, has been generated previously [12]. Based on the similarity in the *Rx* expression pattern in Japanese killifish medaka and mouse [20], a 4-kb DNA fragment upstream of the medaka *Rx3* gene has been identified to contain an evolutionarily conserved region that can direct the *Cre* expression in the mouse retina [12]. However, when we reinvestigated this line we found that beside its activity in optic vesicles, it has a much broader scope of activity and this certain nonspecificity may complicate its use. Here, we have taken advantage of the *Rx* expression and have generated two transgenic mouse lines, *MB31-Cre* and *mRx-Cre,* and compared their recombination potential with that of *Rx-Cre*. Our data demonstrate that among the three analyzed lines, *mRx-Cre* represents an ideal tool for gene manipulation in early retinal progenitors as judged by its specificity, strength and early onset.

## Results and Discussion

**Figure 1:**
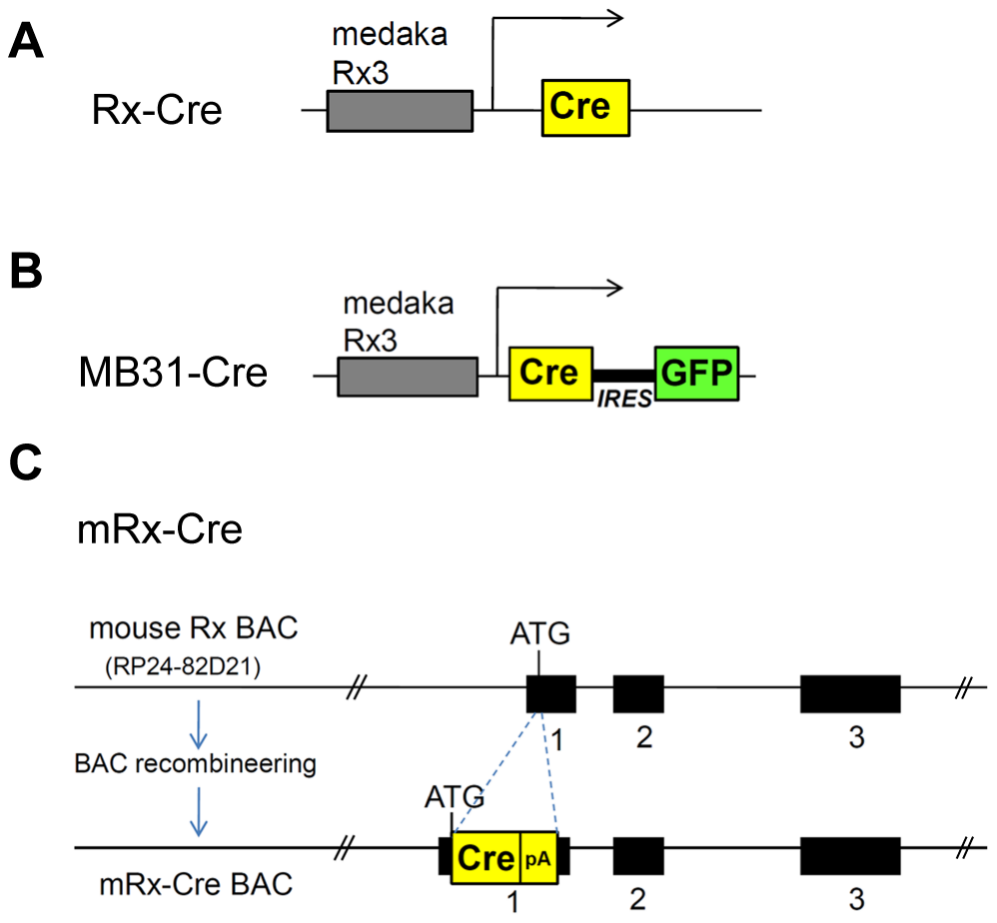
Schematic representation of *Cre* recombinase-expressing transgenic mouse lines used in this study. (A) The *Rx-Cre*; with a 4-kb DNA fragment upstream of the medaka *Rx3* gene driving *Cre* expression [12]. (B) To generate *MB31-Cre*, the 4-kb DNA fragment upstream of the medaka *Rx3* gene was linked to the coding region of *Cre* recombinase. The *IRES* sequence was used to connect *Cre* expression with EGFP to monitor the transgene expression. (C) To generate *mRx-Cre BAC*, a BAC containing 200 kb covering the *Rx* locus was modified by BAC recombineering. The *Cre* coding region (Cre-pA) was inserted into the *Rx* translational initiation start site (ATG). The exons are indicated with black boxes.

**Figure 2:**
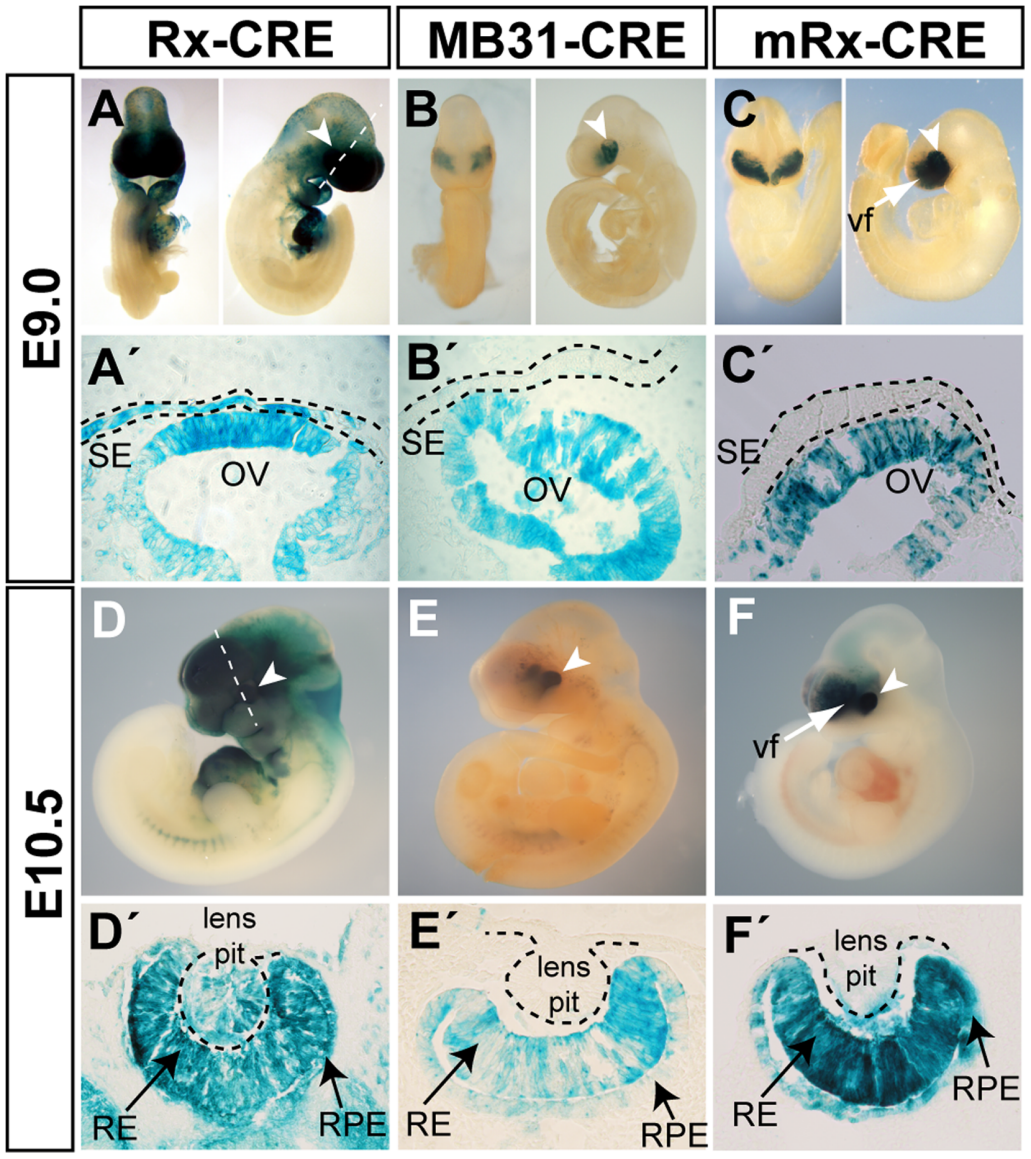
*In vivo* activity of *Rx-Cre*, *MB31-Cre* and *mRx-Cre* transgene products assessed using the *ROSA26R* line. Whole-mounts (A–F) or coronal sections (A’–F’) were stained with X-gal at indicated stages to show the Cre activity in the eye primordium (white arrowheads). Surface ectoderm and developing lens are indicated with dashed lines. SE – surface ectoderm; OV – optic vesicle; RPE – retinal pigmented epithelium; RE – retina; vf – ventral forebrain.

**Figure 3:**
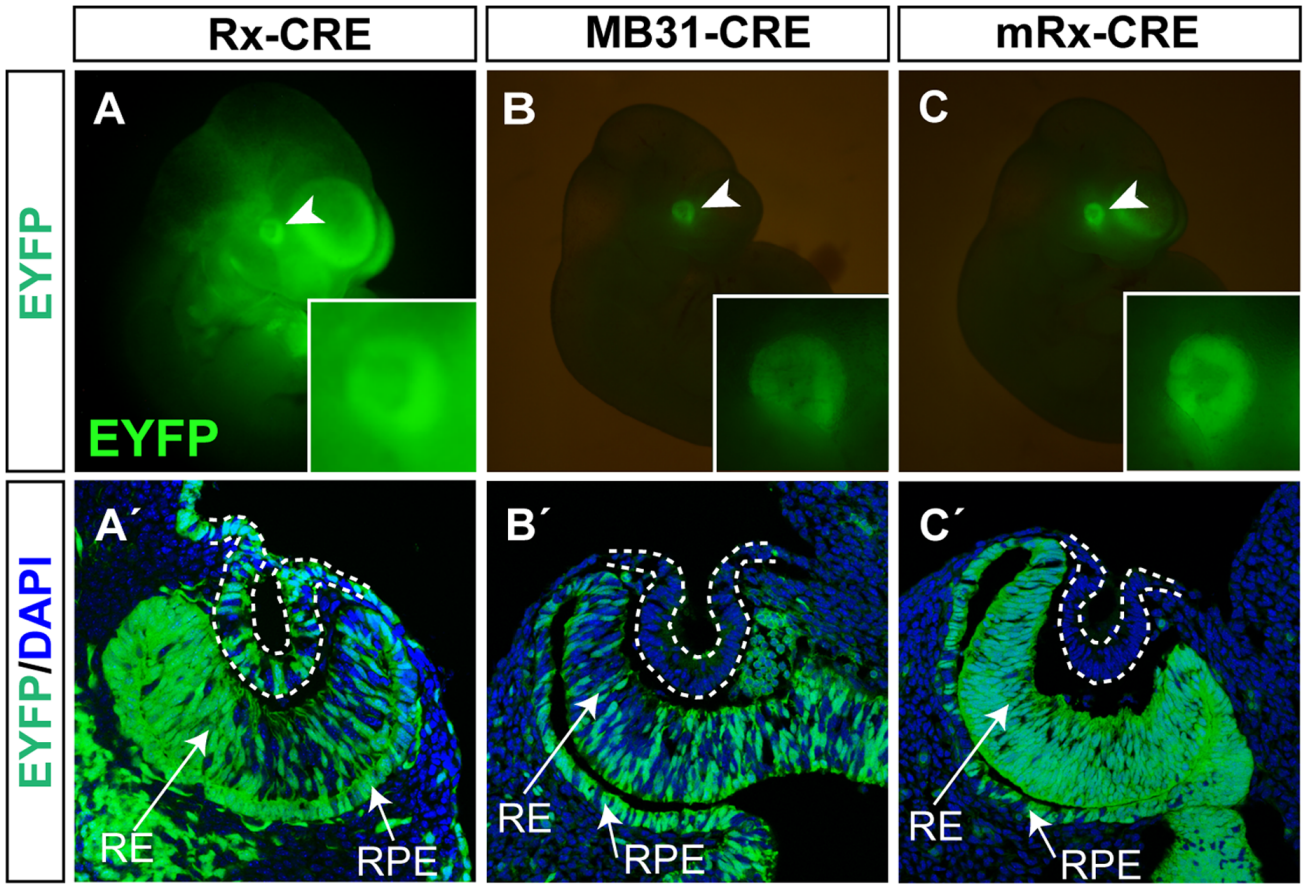
Activity of *Rx-Cre*, *MB31-Cre* and *mRx-Cre* in the eye primordium analyzed using the *ROSA26R-EYFP* reporter line. (A–C) Whole-mounts showing *EYFP* expression (green) in the overall embryo at E10.5. (A’–C’) Coronal sections through the eye region co-stained with DAPI (blue) showing Cre activity in the retina, retinal pigmented epithelium and invaginating lens pit (dashed line) at E10.5.

In order to perform gene inactivation specifically in retinal progenitors of the optic vesicle we first reinvestigated the previously generated *Rx-Cre* [12]. The *Rx-Cre* transgene is schematically depicted in Figure 1A. To define the kinetics and pattern of the Cre recombinase activity driven by *Rx-Cre*, transgenic mice were bred with the *ROSA26R* reporter mouse strain to generate *Rx-Cre; ROSA26R* double transgenic animals. These mice enabled detection of the Cre activity and lineage tracing of *Cre*-expressing cells using X-gal staining. Upon Cre recombination, the expression of *LacZ* under the ROSA promoter is activated by the removal of a stop cassette [21]. The *Rx-Cre; ROSA26R* embryos reproducibly showed a broad area of recombination (Figure 2A,D). Already at E9.0 X-gal staining was observed both in OV and in the overall head region, also targeting head surface ectoderm (Figure 2A,A’). By E10.5 expression was detected in the neuroepithelium of the optic cup and also in the invaginating structure of the lens pit (Figure 2D’). At the early stages of vertebrate eye development, some genes are expressed in both SE and OV that interact and induce their mutual development. To study such genes it is therefore essential to perform gene inactivation in both tissues separately in order to dissect their cell autonomous functions. For this reason, Cre activity in the ectodermal compartment can be undesirable. We thus analyzed recombination in ectoderm-derived structures in more detail using the *ROSA26R-EYFP* reporter line [22]. The Cre-mediated recombination in *ROSA26R-EYFP* results in the expression of fluorescent protein *EYFP* which enables a better signal resolution at the single cell level than *ROSA26R*. The analysis of *Rx-Cre; ROSA26R-EYFP* embryos confirmed Cre activity in SE and invaginating lens vesicle (Figure 3A,A’). Expression of *EYFP* also offered the opportunity to determine the degree of mosaic recombination based on the proportion of EYFP^+^ and DAPI^+^ retinal progenitor cells. The pattern of *EYFP* expression revealed that at E10.5 a considerable amount of cells residing in the retina escaped to *Rx-Cre*-mediated deletion (Figure 3A’).

To establish a genetic tool for directing gene inactivation selectively to the OV compartment, we decided to generate a new *Cre*-expressing line. We employed the same strategy as Swindell *et al* [12], having in mind that a nonspecific expression pattern of the short transgene *Rx-Cre* may depend on its integration site into the genome. The 4-kb DNA fragment upstream of the medaka *Rx3* gene was used to drive the expression of *Cre* and generate *MB31-Cre* (Figure 1B). The coding sequence for fluorescent protein EGFP was linked to *Cre* via the *IRES* sequence for monitoring the recombinase expression. Pronuclear injection yielded nine transgenic founders with three animals exhibiting Cre activity specifically localized in the retina (not shown). One founder was chosen for further analysis using *ROSA26R* and *ROSA26R-EYFP* reporter lines. We were able to detect Cre activity in *MB31-Cre; ROSA26R* embryos as early as at E9.0, specifically in the OV compartment (Figure 2B,B’). At E10.5, as the optic vesicle had invaginated to form the optic cup, the X-gal^+^ progeny of *Cre*-expressing cells contributed to cells in the retina and retinal pigmented epithelium (Figure 2E,E’). In all analyzed embryos, no Cre activity was observed in ectodermal derivatives such as SE or lens pit or in other parts of the embryo (Figure 2B,E). This remarkable specificity was also demonstrated using *MB31-Cre; ROSA26R-EYFP* transgenic embryos, where EYFP was specifically localized in the retinal tissue (Figure 3B) and no EYFP^+^ cells were observed in the invaginating lens pit at E10.5 (Figure 3B’). These results indicate that *MB31-Cre* directed the recombinase activity specifically to the developing retina. However, it should be noted that at E10.5, in both *MB31-Cre; ROSA26R* and *MB31-Cre; ROSA26R-EYFP* embryos, Cre-mediated deletion did not target the whole progenitor cell population since not all retinal cells were X-gal^+^ or EYFP^+^, respectively (Figure 2E’ and Figure 3B’). Although the 4-kb DNA fragment upstream of the medaka *Rx3* gene directed the *MB31-Cre* transgene expression specifically to the retina, it did not reconstitute the expression with regard to the onset and strength observed in the mouse *Rx* gene expression [14]. This may be attributed to the differences in the regulation of *Rx* expression in medaka and mice or to the insufficiency of the 4-kb fragment to cover all the regulation of *Rx*. It is possible that additional *cis*-regulatory elements are required for proper spatiotemporal expression of the *Rx* gene in mice. To circumvent these problems we decided to generate another *Cre*-expressing line, referred to as *mRx-Cre,* in which the *Cre* expression is driven by mouse *Rx* gene regulatory sequences.

**Figure 4:**
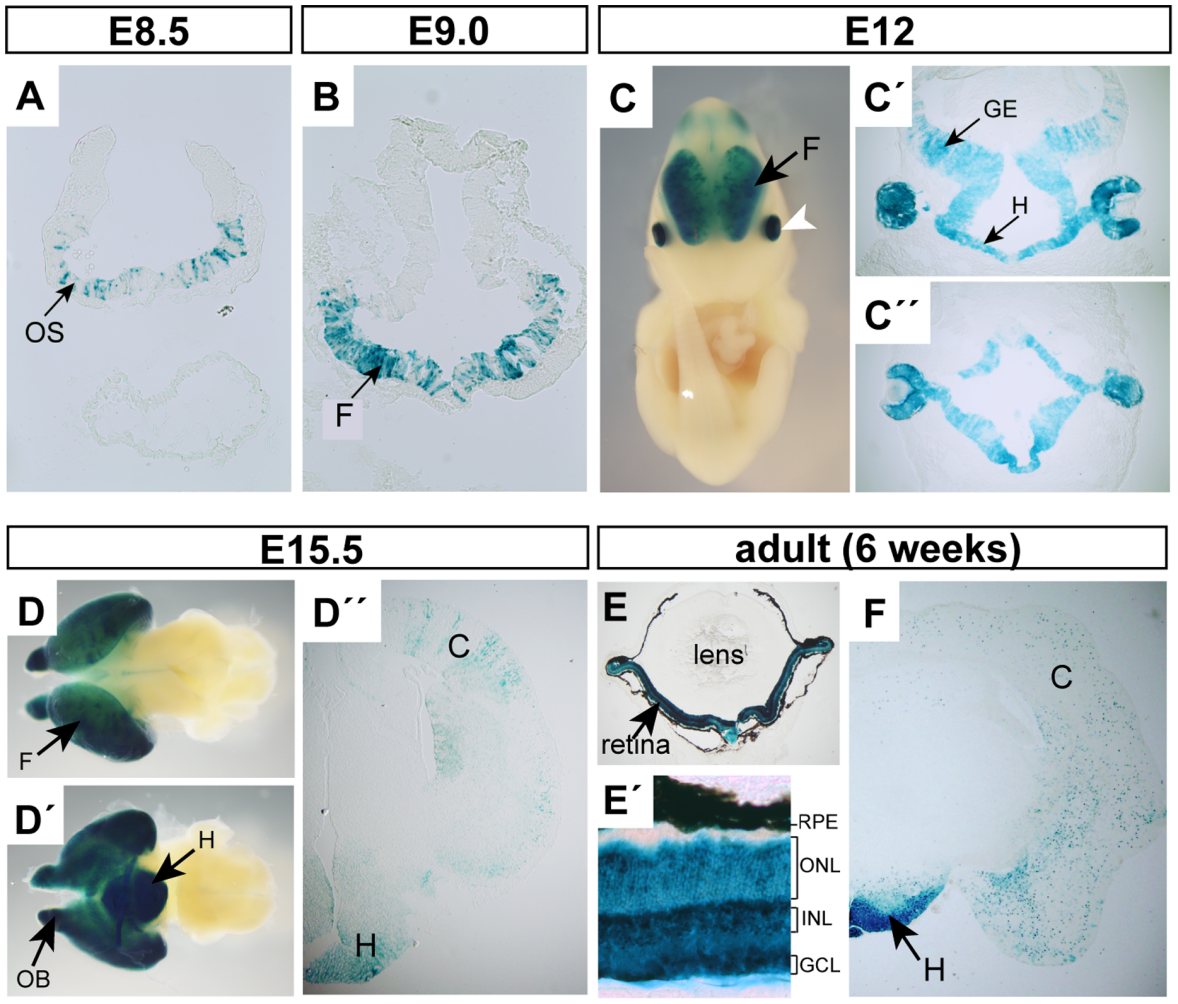
The *mRx-Cre* activity in the eye, forebrain and hypothalamus analyzed using the *ROSA26R* reporter line. Whole-mounts or sections were stained with X-gal at indicated stages to show the *mRx-Cre*-mediated Cre activity. (A) The X-gal^+^ cells were first observed in the optic sulcus of E8.5 embryo. (B–D’) The Cre activity in developing brain. Whole-mounts (C, D, D’), coronal sections (C’, D’) and transversal section (C’) showing Cre activity in embryonic brain. (F) Coronal section of adult brain showing Cre activity in the hypothalamus and cortex. (E–E’) Sections through the adult eye showing strong uniform Cre activity in all layers of the retina. OS-optic sulcus; F-forebrain; GE-ganglionic eminences; H-hypothalamus; OB-olfactory bulbs, C-cortex; RPE-retinal pigmented epithelium; ONL-outer nuclear layer; INL-inner nuclear layer; GCL-ganglion cell layer.

To generate *mRx-Cre* transgenic mouse we selected a BAC clone (RP24-82D21) containing the entire mouse *Rx* gene as well as 95 kb upstream of the *Rx* translational start site and 100 kb downstream of the locus. We employed the method of BAC recombineering [23] to insert the *Cre* recombinase coding sequence into the first ATG of *Rx* gene (Figure 1C). As the 200-kb BAC clone is supposed to carry all *cis*-regulatory sequences ensuring proper spatiotemporal expression, the expression pattern of *Cre* recombinase should imitate that of the endogenous *Rx* gene. The *Cre*-inserted BAC was used for pronuclear injection to generate *mRx-Cre* transgenic mice. We obtained three founders showing expression in the developing retina (not shown). One of them was chosen for further analysis using the *ROSA26R* reporter line. As already mentioned, endogenous *Rx* expression starts between E7.5 and E8.0 [14]. In agreement, we observed the mRx-Cre activity already from E8.5 in the optic sulcus, albeit with a frequent appearance of mosaic recombination (Figure 4A) of *mRx-Cre; ROSA26R* embryos. This slight delay can be explained by a short intermission between the *Cre* expression onset and recombination seen in *ROSA26R*. Consistent with the expression pattern of the endogenous *Rx* gene [14], strong X-gal staining was observed in the optic vesicles of E9.0 *mRx-Cre; ROSA26R* embryos (Figure 2C). Strikingly, at E10.5, the X-gal^+^ progeny of *Cre*-expressing cells contributed to all cells of the retina and the majority of retinal pigmented epithelium cells (Figure 2F’). Therefore, *mRx-Cre* directed Cre activity in all optic vesicle derivates, mainly to the forming retina. This almost absolute recombination rate was further confirmed in *mRx-Cre; ROSA26R-EYFP* eyes at E10.5 showing that virtually all retinal progenitor cells were EYFP^+^ (Figure 3C’). The observation that *mRx-Cre* targets all retinal progenitors in the early stages of eye development was further documented by uniform X-gal staining of all cellular layers of the *mRx-Cre; ROSA26R* adult retina (Figure 4E,E’). Importantly, no EYFP^+^ cells were observed in the invaginating lens pit of *mRx-Cre; ROSA26R-EYFP* embryos (Figure 3C’). In addition, no cells showing recombination were observed in other parts of the embryo than eye, ventral forebrain and hypothalamus. Beside strong β-galactosidase activity in OV-derived structures, a strong although mosaic activity was found in the ventral part of forebrain and in prospective hypothalamus (Figure 4B–D’). Although strong X-gal staining was observed in E15.5 hypothalamus and forebrain after whole-mount staining (Figure 4D–D’), sectioning of E15.5 and adult brains revealed that the *mRx-Cre* activity in the forebrain/cortex is strongly mosaic (Figure 4D’,F). In contrast, the rate of recombination in the hypothalamus appeared very high and we propose that this driver line may be used for genetic studies in the hypothalamus.

In conclusion, we have generated two *Cre*-expressing transgenic mice based on *Rx* regulatory sequences and provided their comparison with previously reported and widely used *Rx-Cre* [12]. In our hands, *mRx-Cre* appears the optimal Cre-driver for retinal progenitors, exhibiting very early onset, strength, specificity, and very low degree of mosaicism. Furthermore, *mRx-Cre* provides a useful tool for the studies of molecular mechanisms facilitating the interaction between OV and SE since it shows selective activity in OV only.

## Materials and Methods

### Ethics Statement

Housing of animals and *in vivo* experiments were performed after approval by the Animal Care Committee of the Institute of Molecular Genetics (study ID#174/2010) and in compliance with national and institutional guidelines (ID#12135/2010-17210).

### Mouse Lines

***mRx-Cre.*** A 200-kb Bacterial Artificial Chromosome (BAC) (RP24-82D21) harboring all coding exons, 5′ and 3′ region of the mouse *Rx* gene was purchased from Children’s Hospital Oakland Research Institute. To generate *mRx-Cre* BAC, the open reading frame of Cre recombinase was inserted into the exon 1 containing the translation initiation codon of *Rx* using a method of BAC recombineering [23]; http://web.ncifcrf.gov/research/brb/protocol.aspx). The recombineering construct pCS-Cre-FRT-neo-FRT was generated by replacing the *DsRed* coding sequence in pCS2+DsRed+FRTKanFRT (provided by James D. Lauderdale [24]) by the *Cre* coding sequence. The Cre-FRT-kan-FRT targeting cassette was PCR-amplified from pCS-Cre-FRT-neo-FRT using *Rx* forward and reverse targeting primers: mRxCreF: 5′-AGGGAACCGGGCATCGAGCTCCAGTTTGCAAAGTGCACTCCCTCCTCACCATGTCCAATTTACTGACCGTACA-3′; mRxCreR: 5′CTTGGTAAAGCCCAGGATGGCTTCGA TGCTGTGCAAACGCGACGTCTCTATTCCAGAAGTAGTGAGGAG-3′. The PCR product was purified using Qiaex Gel extraction Kit (Qiagen) and treated with *Dpn*I to dispose of the template plasmid backbone. The PCR product was then electroporated into RP24-82D21 BAC-carrying bacterial strain EL250, and double resistant colonies (Cm^r^, Kan^r^) were tested for homologous recombination by PCR (primers: F: 5′-AGCACCAAAGCTCCAGTTACC-3′; R: 5′-CGTTGCATCGACCGGTAATGCA-3′). The kanamycin resistance cassette was further removed by induction of *flipase* activity in EL250 cells and the colonies were tested for kanamycin sensitivity. Modified *mRx-Cre* BAC was isolated, treated with *Not*I and applied to a Sepharose 4B-CL column to remove the BAC backbone according to the protocol (http://www.med.umich.edu/tamc/BACcol.html). Fractions were collected and insert integrity was analyzed using pulsed field gel electrophoresis. DNA was used for pronuclear injection. Injection gave us three founders one of which was chosen for further analysis. Mice were genotyped using primers that recognize the recombination junction, with forward primer located upstream of the *Rx* translation start site (F: 5′-AGCACCAAAGCTCCAGTTACC-3′) and reverse primer located in *Cre* recombinase (R: 5′-CGTTGCATCGACCGGTAATGCA-3′). For analysis of Cre activity, the eighth generation from the original founder was used for the generation of presented embryos. For stages E8.5–E10.5, twenty embryos from three to five independent litters were used. For stages E12–E15.5, eight embryos from three litters were used. For adult stages (6 weeks), tissues from three independent animals were used. All embryos (tissues) reproducibly exhibited the same expression pattern with highly comparable strength. Presented expression pattern was stable from F1 generation.

***MB31-Cre.*** The coding region of Cre recombinase was cloned into pIRES2-EGFP (Clontech) and the Cre-IRES-EGFP cassette was fused to the medaka *Rx* gene promoter (provided by Jochen Wittbrodt). DNA was used for pronuclear injection and nine founders were analyzed for activity using *ROSA26R* mice. The least mosaic line, designated *MB31*, was characterized further. For analysis of Cre activity, the fifteenth generation from the original founder was used for the generation of presented embryos. For stages E9.5 and E10.5, twenty embryos from three to five independent litters were used. All embryos reproducibly exhibited the same expression pattern with highly comparable strength.

The *Rx-Cre* mice were described previously and were provided by Milan Jamrich [12]. For analysis of Cre activity, fifteen embryos from three litters were used. All embryos exhibited comparable expression pattern. Both *Rx-Cre* and *MB31-Cre* were genotyped using primers: F: 5′-CATTTGTGAAGTGCTTGAAGGAAT-3′; R: 5′-AGAGGAAGGCAGCACTGATGAAA-3′.

Generation of *ROSA26R* (stock no. 003309) and *ROSA26R-EYFP*, both purchased from Jackson laboratory, was described previously [21], [22]. The genotype was determined by PCR analysis of genomic DNA obtained from tail biopsies.

### Tissue Collections and Histology

Mouse embryos were harvested at several developmental stages from timed pregnant females. The morning of vaginal plug was considered as embryonic day 0.5 (E0.5). Embryos were fixed in 4% paraformaldehyde (w/v) on ice for time depending on embryonic stage (from 20 minutes up to 2 hours). Embryos were washed several times with cold PBS, cryopreserved by overnight incubation in 30% sucrose (w/w), frozen in OCT (Tissue Tek, Sakura Finetek) and sectioned.

### X-Gal Staining

For β-galactosidase assay, embryos were fixed on ice in 0.4% formaldehyde (w/v) in PBS, washed 3×20 minutes with the rinse buffer (0.1 M phosphate buffer pH 7.3, 2 mM MgCl_2_, 20 mM Tris pH 7.3, 0.01% sodium deoxycholate, and 0.02% Nonidet P-40) and incubated in X-Gal staining solution (rinse buffer supplemented with 5 mM potassium ferricyanide, 5 mM potassium ferrocyanide, 20 mM Tris pH 7.3, and 1 mg/ml X-gal) overnight at room temperature. For sectioning, embryos were re-fixed in 4% parformaldehyde (w/v), washed with PBS, cryopreserved in 30% sucrose (w/w) and embedded in OCT (Tissue Tek, Sakura Finetek).

### Immunohistochemistry

For observation of *EYFP* expression, the cryosections were permeabilized with PBT (PBS with 0.1% Tween-20) for 15 minutes, washed 3×10 min with PBT, stained 10 min with DAPI (1 µg/ml) in PBT and mounted into Mowiol (Sigma).
